# Development of a Patient Decision Aid to Prevent Firearm Suicide Among US Women Reserve and National Guard Veterans

**DOI:** 10.1007/s11606-025-09942-4

**Published:** 2025-11-13

**Authors:** Anne G. Sadler, Michelle A. Mengeling, Brian L. Cook, James C. Torner, Mark Vander Weg, Alison B. Hamilton, Jonathan Platt, Amanda Heeren, Lindsey Fuhrmeister, Jeffrey L. Smith

**Affiliations:** 1 VHA Office of Rural Health Veterans Rural Health Resource Center (VRHRC), VA Iowa City Health Care System, Iowa City, IA USA; 2 Center for Access & Delivery Research and Evaluation (CADRE), VA Iowa City Health Care System, Iowa City, IA USA; 3 Department of Psychiatry, University of Iowa Carver College of Medicine, Iowa City, IA USA; 4 Department of Internal Medicine, University of Iowa Carver College of Medicine, Iowa City, IA USA; 5 VA Iowa City Health Care System, Iowa City, IA USA; 6 Department of Epidemiology, University of Iowa College of Public Health, Iowa City, IA USA; 7 Departments of Neurology, Surgery, and Neurosurgery, University of Iowa Carver College of Medicine, Iowa City, IA USA; 8 Department of Community and Behavioral Health, University of Iowa College of Public Health, Iowa City, IA USA; 9 VA Center for the Study of Innovation, Implementation & Policy (CSHIIP), VA Greater Los Angeles Healthcare System, Los Angeles, CA USA; 10 Department of Psychiatry and Biobehavioral Sciences, David Geffen School of Medicine, University of California Los Angeles, Los Angeles, CA USA; 11 School of Social Work, University of Iowa, Iowa City, IA USA; 12 VA Behavioral Health Quality Enhancement Research Initiative (QUERI), Central Arkansas Veterans Healthcare System, Little Rock, AR USA; 13 VA Center for Mental Healthcare & Outcomes Research (CeMHOR), Central Arkansas Veterans Healthcare System, Little Rock, AR USA; 14 University of Iowa Hospitals and Clinics, Iowa City, IA USA

**Keywords:** women veterans, firearm safety, suicide prevention, shared decision-making, patient decision aid

## Abstract

**Background:**

Firearms are the leading method of suicide death among women Veterans, accounting for nearly half of such deaths. Interventions addressing firearm suicide prevention for women Veterans are under-studied.

**Objective:**

To develop a patient decision aid (PtDA) tailored for Reserve and National Guard (RNG) women Veterans to promote safe firearm storage and suicide prevention through informed decision-making.

**Design:**

Multi-phase mixed-methods study including qualitative interviews, semi-structured surveys.

**Participants:**

86 stakeholders: 60 women Veterans, 26 providers.

**Approach:**

Phase 1 qualitative interviews obtained RNG women Veterans’ (n = 35) and providers’ (n = 26) preferences and recommendations for conversations about firearm suicide risk mitigation. Phase 2 surveyed members of a women Veterans engagement group about the PtDA prototype’s acceptability and utility. Phase 3 interviewed RNG women Veteran gun owners (n = 20) about their satisfaction with using the PtDA alone or with shared decision-making (SDM).

**Key Results:**

Phase 1 findings informed the PtDA prototype development, refined in Phase 2. In Phase 3, Veterans reported high satisfaction with the PtDA’s information, tone, and preventative approach. Most agreed Veterans could complete the PtDA safety plan without provider assistance. Nearly half (9/20; 45%) reviewing the PtDA using SDM reported having loaded guns accessible at all times. Most (14/20; 70%) indicated SDM made them more likely to identify and act on a safety plan than if they received the PtDA alone. Nearly all (19/20; 95%) agreed VA providers should routinely use SDM when discussing firearms and suicide risk. All would recommend SDM to other Veterans. Most (19/20; 95%) indicated they would follow their plan to talk with someone about holding their firearms. All indicated they would hand off their firearm if their suicide risk escalated.

**Conclusions:**

Veterans’ high satisfaction with this PtDA indicates its potential to encourage firearm safety planning, engage support, and foster firearm safety conversations to prevent suicide among women Veterans.

**Supplementary Information:**

The online version contains supplementary material available at 10.1007/s11606-025-09942-4.

## INTRODUCTION

Firearm suicide has increased in the USA over the past two decades, now accounting for six in ten gun-related deaths in 2023.^1,2^ Among women Veterans, this crisis is especially pronounced; their firearm suicide rate is nearly 1.5 times higher (144.4%) than that of non-Veteran women—a disparity more than twice that seen between male Veterans and their non-Veteran counterparts (69.9%).^3^ Firearms are the leading cause of suicide death, involved in nearly half (44.4%) of all suicides among women Veterans.^3^ Despite these stark figures, interventions specifically tailored to address firearm suicide among women Veterans remain under-studied.

Notably, many military members and Veterans who attempt suicide have no prior mental health diagnosis,^4–6^ e.g., due to the stigma of seeking mental health care, low perceived need for treatment, and other barriers.^7^ This highlights the critical role of non-mental health care providers in preventing suicide, with opportunities to intervene across the continuum of suicidality, from ideation to attempts. A systematic review reported that, on average, 45% of individuals who died by suicide had contact with primary care within 1 month prior to their death.^8^ Similar findings have been observed among Veterans receiving care through Veterans Health Administration (VHA) primary care.^9^ A national study of suicide decedents found that women who died by firearms were less likely to seek mental health treatment.^10^

Whether they receive care through VHA or elsewhere, women Veterans are more likely than non-Veteran women to experience suicidal ideation and repeated suicide attempts.^11,12^ They are also more likely than male Veterans to contact the Veterans Crisis Line, screen positive for suicide risk, receive a higher suicide risk rating, and be referred to a VA suicide prevention coordinator,^13^ but less likely than men to accept this referral.^14^

Access to firearms is associated with increased suicide risk.^15^ Firearm lethal means safety planning, which focuses on increasing time and space between a person in crisis and access to their firearm, is an evidence-based suicide prevention strategy.^16^ This approach is particularly urgent given that nearly half of suicide attempts happen within 20 min of initial suicidal thoughts^17–19^ and most firearm-related attempts are fatal.^20^ Yet, community and VHA providers do not routinely screen for firearm access or engage in safety planning discussions due to their limited training, uncertainty about its effectiveness and legality, time constraints, lack of decision supports, and belief that such conversations are unnecessary.^21–27^ Even lower rates of provider documentation of firearm access screening have been found among women compared to men Veteran patients in their first year of VHA care, although the study did not report reasons for this disparity.^24^

Low screening rates are a particularly relevant concern for Reserve and National Guard (RNG) Veterans, whose suicide risk is elevated in the first 2 years after leaving the military and who face multiple stressors associated with increased risk, e.g., military traumas, rapid transitions into civilian life, and identity and social support readjustment.^28^ RNG Veterans comprise approximately 38% of the US armed forces. They transition between active military service—including deployments that may confer Veteran status—and civilian life, where health care systems may not consistently recognize them as Veterans.^28,29^ These transitions can create gaps in care, which are particularly concerning for women Veterans, as they are more likely than non-Veteran women to own firearms. Like male Veterans and the general US population, women cite self-protection as the primary reason for gun ownership.^1,30,31^ Women Veterans frequently keep firearms accessible, loaded, and unlocked,^30–32^ a practice associated with significantly increased suicide risk. Firearm owners who store their guns locked and/or unloaded are at least 60% less likely to die by firearm suicide than those who do not.^20^

Women Veterans also experience overlapping and interacting morbidities and stressors, including chronic illness, pain, trauma exposure, mental health disorders, and interpersonal violence.^33^ US suicide death analyses show precipitating events often co-occur across health, relational, and social domains,^34^ underscoring women Veterans’ elevated suicide risk when firearms are accessible and the need for prevention strategies that address trauma exposure and motivations for protection. Existing firearm-related suicide interventions remain sex-neutral, without consideration of the distinct perspectives and needs of women Veterans.^35^

Patient decision aids (PtDAs)^36^ may offer a promising strategy to support safer firearm storage and suicide prevention. PtDAs are evidence-based tools that support people by “making their decisions explicit, providing information about options and associated benefits/harms, and helping clarify congruence between decisions and personal values.”^37^ When applied to firearm means safety, PtDAs can help Veterans make safety choices aligned with their individual values and circumstances, potentially reducing their suicide risk while respecting their rights and autonomy.^.^Additionally, PtDAs may mitigate concerns among women Veterans and providers that firearm-related discussions could cause unintended harm or disrupt therapeutic relationships, by treating them as a routine, preventative health matter.^22,25–27,38^

PtDAs play a pivotal role in supporting shared decision-making (SDM), a collaborative process whereby patients and providers work together to consider options and make health-related decisions.^39^ Recent guidance emphasizes the importance of providers developing cultural competence around firearms and presenting risks and protective factors related to gun safety neutrally and nonjudgmentally to support SDM.^38^ This approach is directly advanced by PtDAs designed to empower Veterans through patient-centered care.

The objectives of this research were to (1) develop a PtDA, using international standards and stakeholder input, to support women RNG Veterans in understanding their risk for firearm suicide and to consider firearm lethal means safety options; and (2) iteratively alpha test the PtDA with women Veterans to assess its acceptability, usability, and perceived value when used independently or when paired with a SDM process. We also sought to evaluate if the PtDA activated Veterans to complete a firearm safety plan and discuss it with their providers or other trusted individuals. This work was grounded in the patient-centered care model, which emphasizes personal agency—the ability of individuals to make informed choices.^41^ This work was also guided by the Health Belief Model, which posits that preventive action occurs when individuals recognize their risk and its seriousness, perceive the benefits of taking action as outweighing barriers, and feel confident in acting and receive cues to act.^42^

## METHODS

This program of research used mixed qualitative and quantitative methods consisting of three phases (7/7/21–4/18/23), involving 86 stakeholder interviews (60 women Veterans, 26 providers). It was funded by the VHA Office of Rural Health and approved by University of Iowa IRB (IRB #201911171). Participants received study information including informed consent elements; consent was implied by participation, per IRB approval.

### Phase 1: Patient Decision Aid Development

**Figure 1:**
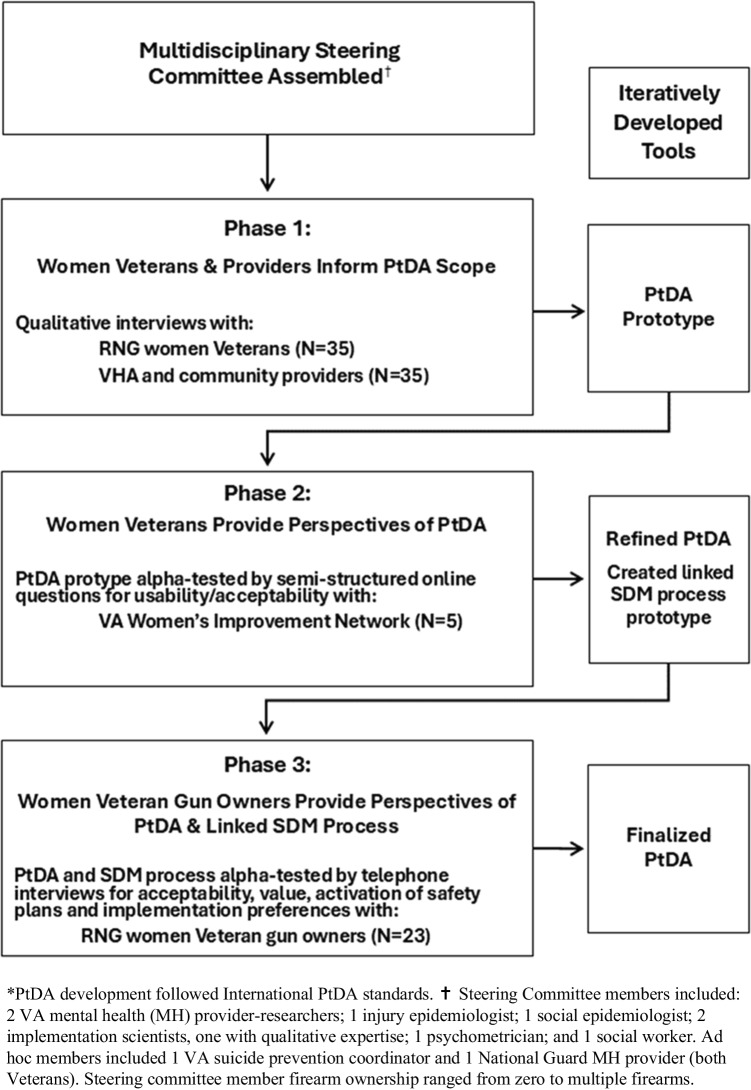
Patient Decision Aid (PtDA) Development Process*.

A national ten-member multi-disciplinary Steering Committee developed and iteratively refined a prototype PtDA focused on firearm lethal means safety planning for women Veterans. Committee members had extensive expertise in military women’s occupational health, trauma-informed care, military culture, service-type, and women-centric approaches to engage and educate Veterans (Fig. 1).

*PtDA development followed International PtDA standards. ✝ Steering Committee members included: 2 VA mental health (MH) provider-researchers; 1 injury epidemiologist; 1 social epidemiologist; 2 implementation scientists, one with qualitative expertise; 1 psychometrician; and 1 social worker. Ad hoc members included 1 VA suicide prevention coordinator and 1 National Guard MH provider (both Veterans).  Steering committee member firearm ownership ranged from zero to multiple firearms.  Guiding the PtDA evolution, the Ottawa Decision Support Framework (ODSF)^43^ emphasizes preparing and supporting patients and providers to use SDM with “difficult” decisions involving multiple options that patients might value differently.^44^ Consistent with the ODSF, we adhered to International Patient Decision Aid Standards (IPDAS) for PtDA development to support a quality decision. We also followed the Standards for UNiversal reporting of patient Decision Aid Evaluation studies^45^ criteria, to ensure transparency in describing our PtDA development process (Appendices 1–2). IPDAS steps include defining the purpose, audience, and scope^46^ of our “Firearm Lethal Means Safety Decision Aid: Options to Prevent Suicide in Reserve and National Guard Women Veterans.”

#### Purpose

To help women Veterans understand their risks of suicide by firearms, make informed choices about their options to prevent it, and assist them in completing a personalized safety plan based on their unique circumstances and preferences, the PtDA focuses actions upstream, prior to precipitating events that might elevate firearm suicide risk. The PtDA is intended to (a) support Veterans’ independent completion of a safety plan without requiring assistance from a provider, and (b) support SDM in discussions with their provider.

#### Audience

RNG women Veterans with access to a firearm.

#### Scope

The scope of the firearm screening and suicide risk mitigation decisional needs and preferences for safety options was informed by current evidence, Steering Committee expertise, and key stakeholders.

#### Veteran Stakeholder Feedback Methods

The VA-DoD Identity Repository identified a community sample of women Veterans residing in Iowa, Arkansas, and California (chosen for racial/ethnic diversity, rurality, and prevalence of post-9/11 RNG Veterans). We over-sampled women Veterans living in rural areas (83%) as they represent a known high-risk population for firearm suicide.^47^ Eligibility criteria included current or prior RNG service; female sex; answering yes to “Do you personally own any guns currently (not including air guns, such as paintball, BB or pellet guns)?”; willingness to be contacted to participate in research; and a valid email. Telephone interviews were completed 5/7/21–6/23/21. Analysis focused on responses to the question: “How would you prefer that a provider screen for or talk with you about guns and suicide?” Veterans received $40 for participation.

#### Provider Stakeholder Feedback Methods

Providers caring for RNG women Veterans were identified by Veteran participants and the VA Women’s Health Practice-Based Research Network, which supports multi-site VA women’s health research.^48^ We recruited 91 community providers by postal mail/phone and 163 VA providers by email and Microsoft Teams messages. Interviews were completed between 12/30/2021 and 7/18/2022. Analysis focused on providers’ responses to, “What approaches have you found work best in engaging your women Reserve or Guard patients in conversations about gun suicide risks and risk mitigation?”

Interviews were completed with women Veterans and providers by experienced qualitative interviewers who participated with the interview guide development. Recorded interviews were transcribed. A rapid qualitative analytic approach^49^ (commonly used in health services research) was used in which two team members independently reviewed and summarized the transcripts, documenting their observations in memos. The analysts then compared and consolidated their summaries and observations, identified themes, and subsequently discussed them with the team to synthesize findings.

#### Phase 1 Findings

Of 81 eligible Veterans, 50 were contacted by email and telephone to reach the 35 completed interviews goal; four (5%) refused and 11 (14%) did not respond. The median age of Veterans interviewed (*n* = 35) was 42 years (range 28–59); most had college and/or graduate/professional degrees (74%); and over a quarter were non-white (29%).

Twelve (13.2%) community and 35 (21.5%) VA providers declined; 75 (82.4%) community and 106 (65%) VA providers did not respond. Twenty-six provider interviews were completed (*n* = 4 community; *n* = 22 VA providers). Providers included four primary care, eight mental health, and 14 specialty care.

Key themes informing the PtDA scope were found to be congruent between Veterans and providers (Table 1), and included the following:

- (1) Gun ownership does not indicate a Veteran is suicidal; (2) misperceptions about firearms and suicide risk persist; (3) providing a straightforward rationale for addressing firearm safety planning is critical; (4) owning guns for self-protection is vital to address in firearm safety planning; and (5) Veteran engagement of trusted individuals is key to lethal means safety planning.

**Table 1:** Phase 1 Qualitative Findings: Exemplar Quotes from Reserve and National Guard (RNG) women Veterans (*N* = 35) and Providers (*N* = 35)* Informing Patient Decision Aid Development

Key themes	Veteran quotes	Provider quotes
Gun ownership is not an indicator that a Veteran is suicidal	“I think that taking an approach that would realize that not just because somebody owns a gun that means they’re suicidal. “	“People have a lot of guns, they know people with guns, family members with guns. Access to arms is higher in rural areas.”
Misperceptions about firearms and suicide risk persist	“When somebody commits suicide it’s usually not like an impulsive thing, it’s something that they’ve been considering.”	“Usually women don’t use them (guns) as often as men.”
Providing Veterans with a straightforward rationale about why providers address firearm safety planning is critical	“Just explaining to them (Veterans), that this is part of our screening process, we just want to make sure that you’re safe.” “The best thing to do is ask them (Veterans) directly, are you thinking of hurting yourself, do you have access to something so you can hurt yourself and what is the plan to remove this object if possible.”	“Have a frank discussion with them (Veterans) about how common suicide risk is. Talk about how firearms (suicide) can be an impulsive act and recommend that they don’t have access to weapons until they feel better.”
Owning guns for self-protection is a vital issue to address in firearm safety planning	“…you know, with the news lately,.., you just never know where you might be when you need to defend yourself so… when I’m out in public, I usually carry. And again, it’s self-defense.”	“For a lot of the female patients, there is a history of trauma, so they see the gun more as a protection than a risk.” “Many feel they need them (guns) to protect their safety.”
Engaging trusted individuals in lethal means safety planning is key	“I mean, giving it to a friend (firearm), have them hold on to the combination” (gun lock) “…the people within their circle of friends have to be really aware, and if they see a situation that doesn’t look right, maybe they can approach the Veteran and say “hey, I’m not feeling like you’re being safe right now, would it be okay if I kept/took your gun?”	“If they are having (suicide) thoughts, have a normative open dialog, it’s not that guns are bad or that I am anti-gun- I just want to educate Veterans on the fact that with guns it’s important to put a barrier between them and the gun.” “Providers often bring in a family member to form a plan to safely remove firearms if there was a need to.”

*Veterans: community sample; providers who serve RNG service members or Veterans: community (*N* = 5), VHA (*N* = 25)

#### PtDA Design

The Steering Committee members integrated their expertise, current research, and Phase 1 findings to inform the key elements of the PtDA prototype (Table 2). It was written in English, at an eighth grade reading level (Flesch-Kincaid Grade),^50,51^ using words, numbers, and visual diagrams to present scientific information. We developed a paper format to better integrate with clinic workflow and to sidestep barriers associated with digital tools (e.g., access, usability, literacy, and privacy risks). Paper decision aids are often viewed by clinicians as easier to embed in practice compared to digital tools.^52^

**Table 2:** Summary of Patient Decision Aid (PtDA) Elements and Goals: *Firearm Lethal Means Safety Plan Options: Reserve and National Guard Women Veterans*

Elements	Goals
**Background/education**	**To inform and activate women Reserve and National Guard Veterans in firearm safety planning** **“This decision aid can assist you in creating your own unique firearm safety plan that can be discussed with your provider or trusted friends and family.”** **“Military members are accustomed to anticipating the unexpected and making contingency plans.’** **“To help you make choices about your best options to prevent suicide with firearms.”**
Purpose of the PtDA	To identify as a decision aid to support informed values-based firearm safety decisions to prevent suicide To promote personal agency in firearm safety decisions To engage Veterans by aligning firearm safety planning with military values of readiness and personal responsibility for safety To promote Veterans’ collaboration with providers and trusted individuals in support of firearm safety
“Why is this important to me as a Reserve or National Guard Woman Veteran?”	To support risk recognition and instill hope that suicide is preventable, while supporting autonomy to take proactive steps to reduce suicide risk
US firearm suicide death statistics	To help women Veterans, who view firearms as a means of safety, contextualize firearm suicide as a health risk for all Americans
Statistics about elevated firearm suicide deaths among women and Reserve/Guard Veterans	To raise awareness in these high-risk Veteran sub-populations and of the unique risks of firearm suicide to activate firearm safety planning among them
Statistics about firearms as the most lethal means of suicide; brief time from thought to suicide action	To emphasize the urgency of upstream, proactive safety planning before a crisis occurs and provide a context for why providers ask about firearm access
Statistics relevant to children in the home and firearms	To raise awareness that firearms are the leading cause of death for children in the US as a context for considering firearm safety
Statistics about frequency of suicidal thoughts among women Veterans	To normalize and validate thoughts of suicide but encourage proactive safety planning
Presentation of suicide myths and evidence that addresses them	To address misconceptions that may prevent engagement in firearm safety planning To increase understanding of how secure gun storage significantly reduces firearm suicide risk
**Integral PtDA Safety Action Plan:** **Key suicide and firearm questions with a decisional checklist and fill in options**	To make options transparent and encourage autonomy and empowerment in navigating safety choices To develop and document a personalized firearm safety plan that can be shared with providers and trusted individuals To increase understanding of how secure gun storage significantly reduces firearm suicide risk To support Veterans’ personal values and situations that inform women’s decisions
PtDA Question #1 topic: Identification of suicide risk factors and symptoms	To raise self-awareness and confidence in recognizing personal risk To become aware of times of increased risk to activate safety plan steps To identify trusted individuals to talk with To identify crisis and routine resources in the VHA, DoD, and community
PtDA Question #2 topic: Identification of what firearms lethal means safety planning is With whom can you share your plan? Consideration of options for: • Routine firearm safety • Temporary firearm removal • When temporary firearm removal is not possible	To reinforce that firearm suicide risk can change over time and that proactive upstream planning allows for personalized safety choices that are aligned with their circumstances and preferences To engage trusted individuals to step up to help with their plan
PtDA #3 Topic: Personal safety when there isn’t a firearm in the home Non-lethal options to consider for ensuring and sustaining safety	To acknowledge personal protection and safety concerns and realities and identify alternatives To support trauma-informed care
**References with electronic links**	To support credibility of information and promote accessibility of resources
Resources for emergencies and routine care resources	To increase awareness of resources across different domains of need To support credibility of information provided in the PtDA and encourage further exploration
**Every page:** Veteran Crisis line logo and contact information	Ensuring visibility and awareness that immediate access to crisis support is available 24/7

#### Phase 2: PtDA Prototype Acceptability and Utility to Women Veterans

We surveyed the VA Women’s Improvement Network (WIN)^53^ with a brief (six items) survey to assess how acceptable and useful they found the PtDA prototype content and approach (alpha testing). The WIN is a virtual national group of women Veterans who provide feedback to researchers and policymakers on initiatives specific to women Veterans (Appendix 3).

#### Phase 2 Findings

Five WIN members provided survey responses by email January 2023. All responded “yes” to each of these questions: (1) “Is this decision aid content potentially useful?”; (2) “Do you think this information could help women Veterans address firearm safety and suicide?”; and (3) “Would you recommend this (decision aid) to a relevant peer?”

Additionally, WIN participants responded to open-ended questions about the acceptability, potential for the PtDA to engage women Veterans in safety planning, and recommendations for implementation (Table 3). All but one indicated they would complete the proactive firearms safety planning supported by the PtDA. That Veteran indicated she would do so with family or provider encouragement. All participants recommended the PtDA be widely available within VA for provider interfaces, waiting rooms, outreach, and appointment mailings; and within Department of Defense (DoD) settings. Suggested improvements included citing additional statistics specific to women Veterans and editorial changes. Notable themes included (a) the importance of the statistics (“Knowledge is power! If you know better you can do better”); (b) the tone (“direct yet caring”); and (c) agreement with its upstream approach (“It’s good to have a plan before you are in crisis”).

**Table 3:** Women’s Improvement Network (WIN) Patient Decision Aid (PtDA) Feedback

Topic	Veteran quotes
PtDA acceptability and ability to engage Veterans?	“It is well organized. I followed the flow. **It is direct yet sounds caring. I appreciate the up-front statistics. It drew me in.”** **“**It is very informative. The way it is presented is good. The tone was excellent. If I was suicidal, I would be willing to go for help.” “I liked the safety plan decisions, options, and questions chart. It was simple to follow if someone was in crisis. The resources give many options on where to go and who to call. When a crisis occurs, you should not have to think about what to do. This document lays out what to do.” **“Knowledge is power! If you know better, you can do better.”** “Surprised at the statistics for women Veterans, sadness, hope with safety plan: 1) factual, anticipatory (plan); 2) I liked the format of statistics side by side with plan and clear concise suggestions for formulating the plan and the multiple options to consider an individual plan; 3) resources to back up statistics; 4) emotionally hard to read (personal experience with family/friends and suicide), but gave me hope that this is a clear way to decrease this response (suicide).” **“**As a retired Safety Officer, I've worked my entire life career in "Risk Management", so at a glance, this was already complete.”
Would you complete the PtDA action plan?	**“**If I was in crisis and working through issues I would use it. If my therapist or loved one asked me to complete it, I would use it **It’s good to have a plan before you are in crisis.”** “I definitely would. It's well organized, informative, and helpful.” “I would fill it out. If I had a family member that would do it with me. I’d probably be more thoughtful with my answers and their help.” “Not sure, I probably would think about the suggestions, and depending on how severe my symptoms were, I might complete some of it. Motivation would be (from) any family/friends/health providers I share it with. If one of the above were aware of my feelings AND encouraged me ‘in the moment’ to complete it, I would be motivated.” “Having had personal experience with family and friends and suicide attempts/completion, this ‘spoke’ to me. I did complete it and think it’s a great way to do proactive counseling for all Veterans especially women, given the rather shocking statistics. The knowledge of these statistics would certainly have given me as a health care provider ‘tools’ to use.”

The Steering Committee responded to WIN recommendations for PtDA improvement by including additional statistics, improving visual presentation, and editorial revisions. Given positive WIN feedback, the committee developed a SDM process interview to accompany the PtDA. This process was guided by the Agency for Healthcare Research and Quality SHARE Model^54^ which uses the PtDA to help Veterans (a) consider firearm safety planning options to decrease their suicide risk; (b) reach decisions on a safety plan; and (c) evaluate satisfaction with their personalized plan.

#### Phase 3: PtDA Acceptability and Satisfaction When Used with a SDM Process Implemented by Study Staff

The next step was to alpha test the refined PtDA using the SDM process interview. The goal was to gather feedback to further refine the tool and inform its feasibility and utility in clinical settings to support SDM. We aimed to understand a community sample of RNG women Veteran gun owners’ perceptions of the PtDA and SDM process and its impact on their firearm safety planning, and suggestions for improvements. International standards emphasize that iterative, patient-centered piloting of PtDAs is critical groundwork for eventual provider uptake and effective SDM. Bypassing this step risks limiting impact by failing to ensure Veterans’ engagement and relevance, which in turn undermines provider adoption.

We re-engaged the sub-sample of Phase 1 community RNG women Veterans who acknowledged gun ownership, updated with current emails. Recruitment was completed by email and telephone (*n* = 107). Participants completing the interview were reimbursed $40 with an additional $10 incentive if they scheduled or completed the interview prior to our recruitment call.

Prior to interview, Veterans were e- and postal mailed the PtDA and asked to review it and have it present for their call. Veterans were informed in the invitation and on the call that this was not a clinical session but a feedback opportunity to evaluate the PtDA’s utility with a SDM process to inform its potential as a model for firearm safety planning. During the call, the PtDA was reviewed with the interviewer, giving the Veteran time to complete their personalized safety plan. Veterans were then asked semi-structured questions about their perceptions of the PtDA and SDM process. Interviewers did not review Veterans’ personalized firearm safety plans with them, as a clinician would.

Of the 107 eligible women Veterans, 30 were recruited, seven declined, 23 consented to participate, and we interviewed the first 20 consenters (the number funded to complete). Telephone interviews were completed by female interviewers 3/27/23 through 4/18/2023. Data were analyzed using descriptive statistics.

#### Phase 3 Findings

The majority of participants (60%) were aged 30–44 years (range 30–59) (Appendix 4); 40% were non-white; and most (70%) had only men for sexual partners over their lifetime. All had completed college or higher education, with 85% currently working. Most (70%) had a current joint family annual income under $100,000, and 40% had children under age 18 living in their household. Most were enlisted or non-commissioned officers (85%), had prior active component military service (90%), and had been deployed to Iraq or Afghanistan post 9/11 (80%).

The gun types that participants owned (not mutually exclusive) included handguns (75%), rifles (45%), and shotguns (40%), with 40% owning three or more guns. When considering gun storage, almost half had their guns loaded and accessible at all times (45%), and over half had guns locked only some of the time or never (60*%).*

##### Satisfaction with Decision Aid and SDM Process

Participants (*n* = 20) reported favorable perceptions of the PtDA and SDM process and implementation preferences (Table 4). Most agreed or strongly agreed that the PtDA helped them to (1) learn new information about the relationship between firearm safety and suicide risk (70%); and (2) better understand suicide risk and options to decrease this risk (65%). Most (80%) believed the information was presented in a way consistent with their choice to have firearms in their home; and most (90%) would recommend the PtDA to other Veterans with firearms in their homes.

**Table 4:** Phase 3 Reserve and National Guard Women Veterans’ Satisfaction with Patient Decision Aid (PtDA) Reviewed Independently and with a Shared Decision-Making Process (*N* = 20)

Question	Strongly agree or agree % (*n*)	Neither agree nor disagree % (*n*)	Strongly disagree or disagree % (*n*)
**Perceived value of PtDA**			
As a result of the (Firearm Lethal Means Safety) decision aid, I learned new information about the relationship between suicide risk and firearm safety	70 (14)	25 (5)	5 (1)
The decision aid information and action steps helped me to better understand suicide risk and think about options for managing my firearms to decrease my risk of suicide	65 (13)	35 (7)	0 (0)
I believe that the decision aid presented information in a way that is consistent with my choice to have firearms in my home	80 (16)	15 (3)	5 (1)
I would recommend the decision aid to other Veterans who have firearms in their home	90 (18)	5 (1)	5 (1)
**Preference for PtDA implementation**			
I believe that Veterans could complete the decision aid action steps (to develop a personal firearm safety plan) on their own without assistance from a VA healthcare provider or suicide prevention coordinator	75 (15)	20 (4)	5 (1)
I believe that the firearm lethal means decision aid tool should be*:			
Made available on the VA website	100 (20)	0 (0)	0 (0)
Given to me by a VA suicide prevention coordinator or nurse care manager and discussed in a call or meeting	100 (20)	0 (0)	0 (0)
Given to me by my mental health care provider and discussed in an appointment	95 (19)	0 (0)	5 (1)
Left in a VA waiting room for Veterans to see	90 (18)	0 (0)	10 (2)
Sent with VA appointment information	70 (14)	10 (2)	20 (4)
Given to me by my primary care provider and discussed in an appointment	65 (13)	20 (4)	15 (3)
**Perceived value of shared decision-making with PtDA**			
I believe that VA providers should routinely use shared decision-making about firearm lethal means safety and suicide risk when they provide care to Veterans	95 (19)	0 (0)	5 (1)
I would recommend using this shared decision-making process about firearm lethal means safety and suicide risk to other Veterans	100 (20)	0 (0)	0 (0)
The firearm lethal means shared decision making process addressed all of the concerns I have about options for managing my firearms to decrease my risk of suicide. (n = 19)	84 (16)	11 (2)	5 (1)
Having my firearm safety action plan which resulted from the shared decision-making process makes me feel safer	70 (14)	30 (6)	0 (0)
I believe that I was more likely to identify my personal action steps and plan to act on them because of the shared decision-making process than if I had received the decision aid tool alone	70 (14)	15 (3)	15 (3)
**Veteran’s planned firearm safety changes resulting from shared decision-making with PtDA**			
As a result of the process of shared decision-making using the decision aid, I will:			
Follow my (firearm safety) action plan to hand off my firearm to a safe party if I need to	100 (20)	0 (0)	0 (0)
Talk with my partner, a family member, friend or someone else about a plan for holding my firearms if I or someone who cares about me had concerns about my safety from suicide	95 (19)	5 (1)	0 (0)

*Other settings Veterans recommended that the firearm lethal means decision aid should be made available in: (1) Veteran-focused organizations, e.g., American Legion, Vet Centers; (2) community settings, e.g., peer supports, emergency response technicians; (3) health care settings, e.g., PTSD groups, mailed with appointment reminders; (4) Department of Defense, e.g., annual briefings, distributed to squadrons and on information boards similar to what we have for disasters

Most agreed or strongly agreed that Veterans could complete the PtDA and develop a personal firearm safety plan without assistance from a VA provider or suicide prevention coordinator (75%). Veterans agreed or strongly agreed that the PtDA should be made available on a VA website (100%); given by and discussed with a suicide prevention coordinator or nurse care manager (100%); initiated in an appointment with a mental health provider (95%); left in waiting rooms (90%); sent with a VA appointment (70%); or provided by/discussed with their primary care provider (65%).

##### SDM Perceived Value

Most Veterans reported favorable perceptions about the SDM process (Table 4). They agreed or strongly agreed that VA providers should routinely use SDM in discussions about firearm lethal means safety and suicide risk when providing care to Veterans (95%); they would recommend SDM to other Veterans (100%); the SDM process using the PtDA addressed all of their concerns about options for managing their firearms to decrease their suicide risk (84%); the firearm safety action plan resulting from the SDM process made them feel safer (70%); and that because of the SDM process, they were more likely to identify their safety plan and act on it than if they had only received the PtDA (70%).

As a result of the SDM process using the PtDA, 100% of participants indicated they would follow their action plan to hand off their firearm to a safe party if needed; and 95% indicated they would talk with a partner, family member, friend, or someone else about a plan for holding their firearms if they or someone who cares about them had concerns about their safety from suicide.

## DISCUSSION

Guided by international PtDA standards and input from key stakeholders across three phases, we developed a PtDA tailored for women RNG Veterans to support informed decision-making and encourage completion of a personalized firearm safety plan. Veterans reported high satisfaction and endorsed the PtDA’s utility both independently and with a SDM process delivered by study staff, underscoring its potential for firearm safety planning and suicide prevention. Positive feedback likely reflects the meaningful involvement of women Veterans and their providers in developing the PtDA, as well as our Steering Committee’s expertise. Findings align with prior research on Veterans’ preferences for firearm safety discussions in primary care, emphasizing care for health and well-being, a nonjudgmental approach, clear rationale for and normalization of the topic, and addressing fears about health system responses as the safety plan is focused on their autonomy.^21–27,55–57^

An important finding is the potential for the PtDA to support meaningful conversations between women Veteran gun owners and health care providers. Participants reported feeling safer when engaging in SDM using the PtDA, and 95% agreed that VA providers should routinely use this approach in clinical care. Importantly, all participants said they would recommend SDM for firearm safety and suicide prevention to other Veterans, and thought the PtDA should be available on a VA website. These results suggest that a tailored, upstream intervention like this PtDA is not only acceptable to women Veterans with firearm access, but also effectively addresses safety concerns and supports firearm safety planning as a part of routine preventive health care. Notably, PtDA use may also help overcome previously identified provider^21–24^, as well as health care system barriers to discussing firearm safety, e.g., tools to engage providers, standardized materials.^55–57^

Another key finding is that using the PtDA with a SDM process may empower women Veterans to proactively involve others in supporting them with firearm safety planning. Nearly all gun owners reported they would discuss a plan for firearm storage if their suicide risk increased, and all participants reviewing the PtDA with SDM indicated they would follow a plan to hand off their firearms to a safe party if needed. Notably, the majority had served in Iraq or Afghanistan post-9/11, and nearly half reported keeping loaded, unlocked guns accessible. Additionally, three-quarters acknowledged having handguns, the most common firearm type used in suicides among US women.^58^ These findings underscore the potential of this approach to facilitate upstream firearm safety planning and engage trusted individuals in interrupting a decision to act on a suicide impulse.

Key factors differentiate our PtDA from previous research and have implications for providers, researchers, and policy makers. First, to our knowledge, this is the first study to develop a firearm lethal means PtDA tailored to and informed by women Veterans. In contrast, “Lock to Live” is a sex-neutral decision aid that addresses lethal means of all types and is not specific to Veterans.^59^ The VA National Center for PTSD “Safety Plan” App also comprehensively addresses lethal means, helping Veterans and non-Veterans “create steps you can take the next time you are in crisis.”^60^ A recent randomized controlled trial testing a digital health tool for patients with suicide risk likewise addressed both firearm and medication storage, but was not tailored to women Veterans.^61^ Second, our PtDA focuses on the military ethos of preparedness that supports Veterans’ personal agency to make safety decisions which is consistent with an upstream prevention approach. Third, the PtDA directly communicates why firearm and suicide screening is needed within a context of risk and protective choices, providing actionable steps to trigger and assist Veterans to develop a personalized safety plan. Fourth, the PtDA uniquely supports decisions about non-lethal means of self-protection, addressing a known barrier to engaging gun owners in firearm safety planning and supporting trauma-informed care for women. Finally, we piloted the PtDA with a SDM process and elicited Veteran perspectives of its acceptability and utility in clinical encounters.

This work is consistent with the VA National Strategy for Preventing Veteran Suicide^62^ focusing on promoting heathy and empowered Veterans, families, and communities. It addresses implementation principles of focusing on not only awareness but also activation to change behaviors of Veterans (and ultimately providers), and in tailoring solutions to at-risk sub-populations instead of a “one-size-fits-all” approach.

This work has limitations and strengths. Our PtDA does not specifically address decisional choices and suicide risk for Veterans who cohabitate with a gun owner or who may later buy a gun. However, it provides important information for any woman Veteran with access to a firearm in a sharable format. Self-selection bias may have increased participation among women gun owners with existing concerns about firearm suicide or who have experienced the loss of a Veteran, military peer, or family member through firearm suicide. If so, these women’s experiences and awareness of the complexity of these fatal choices could contribute knowledge or perspectives benefitting the utility of our findings. Low provider response rate is a limitation. Further, Veteran and provider interviews informing PtDA development were completed approximately 2 years prior to our PtDA alpha testing. It is common for PtDA development to be a lengthy incremental process^63^ and Veterans’ positive feedback indicates that our interview findings remain relevant. The PtDA was not alpha-tested with providers, although we elicited their perspectives to inform its development. Lastly, this work was piloted with RNG Veterans and findings may not be generalizable to all women Veterans. However, most evidence presented, and the decisional choices, are relevant to all women Veterans and 90% had served in the active component military. This work was performed with community samples of Veterans and has potential utility for community outreach and for both VA and community providers.

## CONCLUSION

We developed a PtDA tailored to women Veterans to promote safer firearm storage and advance suicide prevention through informed, patient-centered care. Veterans’ high satisfaction, whether using it alone or with SDM, demonstrates its capacity to strengthen safety planning, involve trusted individuals, and promote respectful firearm safety. Through iterative involvement of Veterans, providers, and other stakeholders, we demonstrated the feasibility and acceptability of integrating firearm safety into routine preventive care. Framing suicide risk as a health concern and offering a patient-centered tool may enable more effective and sustainable prevention for women Veterans, with promise for both VA and community settings and adaptability to other high-risk populations. Future work should test provider use of the PtDA in real-world clinical settings, clinical trials, and development of an electronic format to assess feasibility, usability, and potential for broader implementation.

## Supplementary Information

Below is the link to the electronic supplementary material.

Supplementary Material 1 (DOCX 121 KB)
