# Supplementary material for: Development of a Patient Decision Aid to Prevent Firearm Suicide Among US Women Reserve and National Guard Veterans
Source: J Gen Intern Med. 2025 Nov 13;41(8):2182–94. doi: 10.1007/s11606-025-09942-4 (PMC13241574; doi:10.1007/s11606-025-09942-4)
Supplement: Supplementary file 1 — Supplementary Material 1 (DOCX 121 KB) [file 11606_2025_9942_MOESM1_ESM.docx]

Appendix 1: IPDAS Patient Decision Aid Checklist for Users


Appendix 2: The Standards for Universal Reporting of Patient Decision Aid Evaluation (SUNDAE) Checklist for Evaluation Studies of Patient Decision Aids

| **Section/Topic** | **Page No.** | **Item No.** | **Checklist Item** |
| --- | --- | --- | --- |
| **Title and Abstract** |  |  |  |
|  | 1, | 1 | Use the term patient decision aid in the abstract to identify the intervention evaluated and, if  possible, in the title. |
|  | 2 | 2 | In the abstract, identify the main outcomes used to evaluate the patient decision aid. |
| **Introduction** | **As part of standard introduction (the problem, gaps, purpose)** | | |
|  | 3 | 3 | Describe the decision that is the focus of the patient decision aid. |
|  | 7,9 | 4 | Describe the intended user(s) of the patient decision aid. |
|  | 4-7 | 5 | Summarize the need for the patient decision aid under evaluation. |
|  | 5,7 | 6 | Describe the purpose of the evaluation study with respect to the patient decision aid. |
| **Methods** | **Studies with a comparator should also address Items 7-13 for the comparator if possible** | | |
|  |  | 7 | Briefly describe the development process for the patient decision aid (and any comparator) or cite other documents that describe the development process. At a minimum include: |
|  | 10-17 |  | - The process for gathering, selecting and appraising evidence to inform its content |
|  | 10-17 |  | - Any testing that was done |
|  |  | 8 | Identify the patient decision aid evaluated in the study (and any comparator) by including: |
|  | 9,40 |  | - Name or information that enables it to be identified |
|  | 12,1439 |  | - Date and/or version number |
|  |  |  | - How it can be accessed, if available |
|  | 12 | 9 | Describe the format(s) of the patient decision aid (and any comparator) (e.g. paper, online, video). |
|  | 40-42 | 10 | List the options presented in the patient decision aid (and any comparator). |
|  | X | 11 | Indicate the components in the patient decision aid (and any comparator) including: |
|  | 41 |  | - Explicit description of the decision* |
|  | 41 |  | - Description of health problem* |
|  | 41 |  | - Information on options and their benefits, harms, and consequences* |
|  | 41 |  | - Values clarification (implicit or explicit)* |
|  | 41-43 |  | - Numerical probabilities |
|  | 41-43 |  | - Tailoring of information or probabilities |
|  | 41-43 |  | - Guidance in deliberation |
|  | 41-43 |  | - Guidance in communication |
|  |  |  | - Personal stories |
|  |  |  | - Reading level or other strategies to help understanding |
|  |  |  | - Other components |
|  |  |  | *These components are needed to meet the definition of a patient decision aid. |
|  | X | 12 | Briefly describe the components from Item 11 that are included in the patient decision aid (and any comparator) or cite other documents that describe the components. |
| **Section/Topic** | **Page No.** | **Item No.** | **Checklist Item** |
| **Methods (cont.)** | 8-17 | 13 | Describe the delivery of the patient decision aid (and any comparator) including:   - How it was delivered (e.g. by whom and/or by what method) - To whom it was delivered - Where it was used - When it was used in the pathway of care - Any training to support delivery - Setting characteristics and system factors influencing its delivery |
|  |  |  | Describe any methods used to assess the degree to which the patient decision aid was delivered and used as intended (also known as fidelity). |
|  | 8-17 | 14 | Describe any methods used to understand how and why the patient decision aid works (also known as process evaluation) or cite other documents that describe the methods. |
|  | 7-9 | 15 | Identify theories, models or frameworks used to guide the design of the evaluation and selection of study measures. |
|  |  | 17 | For all study measures used to assess the impact of the patient decision aid on patients, health professionals, organization, and health system: |
|  | 7-9 |  | - Identify the measures |
|  | 7-9 |  | - Indicate the timing of administration in relation to exposure to the patient decision aid and health care interventions |
|  |  |  | For any instruments used: |
|  |  |  | - Name the instrument and the version (if applicable) |
|  |  |  | - Briefly describe the psychometric properties, or cite other documents |
| **Results** | **In addition to standard reporting of results** | | |
|  | 7-9,  53 | 18 | Describe the characteristics of the patient, family, and carer population(s) (e.g. health literacy, numeracy, prior experience with treatment options) that may affect patient decision aid outcomes. |
|  | 10,1439, | 19 | Describe any characteristics of the participating health professionals (e.g. relevant training, usual care vs. study professional, role in decision making) that may affect decision aid outcomes. |
|  | 11-15 | 20 | Report any results on the use of the patient decision aid: |
|  |  |  | - How much and which components were used |
|  |  |  | - Degree to which it was delivered and used as intended (also known as fidelity) |
|  | 11-17 | 21 | Report relevant results of any analyses conducted to understand how and why the patient decision aid works (also known as process evaluation). |
|  |  |  | Report any unanticipated positive or negative consequences of the patient decision aid. |
| **Discussion** | **As part of the standard discussion section (summary of key findings, interpretation, limitations and conclusions):** | | |
|  | 17-21 | 24 | Discuss whether the patient decision aid worked as intended and interpret the results taking  into account the specific context of the study including any process evaluation. |
|  | 17-21 | 25 | Discuss any implications of the results for patient decision aid development, research, implementation, and theory, frameworks or models. |
| **Conflict of Interest** |  |  |  |
|  | X | 23 | All study authors should disclose if they have an interest (professional, financial or  intellectual) in any of the options included in the patient decision aid or a financial interest in the decision aid itself. |

**Appendix 3: VA Women’s Improvement Network (WIN) Member Survey Result Summary. October 2023**

The VA Women’s Health Improvement Network (WIN) Member Survey was disseminated to WIN members in June of 2023 to better understand WIN members engagement preferences and experiences. 14 WIN Members (Veterans) responded.

**Demographics**

Most respondents (71%) reported being between 35-64 years of age, with the largest percentage being between 55-64 years of age (closely followed by those 35-44 years). Most respondents (73%) identified as white; 7% identified as Black or African American, 7% Asian or Asian American, 7% American Indian or Alaska Native and one person declined to respond. In total, 5 states and 8 cities are represented by the respondents.

**Health Care, Volunteerism, and Military Service**

86% of respondents had received health care at the VA in the past year and 71% were affiliated with military or veteran groups outside of the WIN. Some of these groups included Women Marines Association, American Legion, and Veterans Community Response. Most had served in the Army or Reserve/National Guard (68%), although most branches of military were represented (Marines, Air Force, and Navy). Just over one-third (35%) had served from September 11, 2001 to the present, followed by Between the Persian Gulf War and 9/11 (23%).

**Research Interests**

The most common research topics of interest to respondents were women’s health (23%), mental health (20%), physical health (20%), and healthcare access and utilization (18%). Research topics of less interest included post-deployment health and healthcare organization and delivery.

**Areas of VA Healthcare that Need Improvement**

Participants suggested that women Veterans need to feel more welcome within the VHA system. Respondents suggested increasing access to primary care as well as specialty care services. Other suggestions included expanding the availability of women’s only clinics, including staffing all sites with OB-GYN providers. A few participants reported that retention of physicians at the VA as a concern, as it creates potential inconsistency in quality of care.

**WIN Members’ Research Engagement Experiences**

Most respondents (93%) expressed comfort sharing opinions in the WIN and 100% reported trust in fellow WIN members. 100% of respondents stated that they had a lot of respect for other members of the WIN, and 93% reported feeling comfortable communicating openly and discussing different opinions. Respondents felt comfortable sharing because they felt their opinion mattered and their voice was helpful to understanding the experience of Women Veterans. 100% of survey respondents endorsed a clear understanding of WIN’s mission and believe that the greater Veteran community will benefit from WIN’s collaboration with researchers. Regarding collaboration within the group, 86% of respondents felt that what they wanted to accomplish seemed to be the same as other members of the WIN. 86% of respondents felt that the WIN is committed to diversity and 93% felt that the power is shared equally and there is a clear process for making decisions. Most respondents (93%) felt informed about WIN’s engagement activities, noting reasonable time commitment and appropriate compensation. When asked if a sense of trust was experienced in interactions with the WIN facilitators and coordination staff, the survey respondents mentioned feeling respected, acknowledged, and included. When asked how researchers can help to build trust with Veterans who participate in their research, suggestions included staff introductions, active listening, and transparency in explaining the goals of the research and sharing results along the way.

*The WIN is sponsored by the VA Women’s Health Research Network (SDR-10-012)*

Appendix 4. Phase Three Participant Characteristics of Reserve and National Guard (RNG) Women Veterans Who Reported Gun Ownership (N=20)

| Sociodemographic Characteristics | | % (n) |
| --- | --- | --- |
| Age | 30-44 years | 60 (12) |
|  | 45-59 years | 40 (8) |
| Race/Ethnicity | Non-Hispanic White | 66.67 (12) |
|  | Non-Hispanic Black | 25 (5) |
|  | Non-Hispanic Other/Multi | 5 (1) |
|  | Hispanic | 10 (2) |
| Current Marital Status | Currently Married/partnered* | 95 (19) |
| Lifetime sexual partner | Men only | 70 (14) |
|  | Women only | 5 (1) |
|  | Both men and women | 25 (5) |
| Highest Education | Bachelor’s degree or higher | 90 (18) |
|  | Some college or tech training | 10 (2) |
| Currently working | Yes | 85 (17) |
| Current joint family income | < 100K | 70 (14) |
|  | 100K or more | 30 (6) |
| Enrolled in VA care (n=16) | Yes | 70 (14) |
|  | No | 10 (2) |
|  | Don’t Know | 20 (4) |
| VA service-connected disability | Yes | 50 (10) |
| Primary Insurance Coverage | VA coverage | 40 (8) |
|  | Employer/Private/Tricare | 60 (12) |
| Children < 18 years in household | Yes | 40 (8) |
| Rural Residence | Yes | 55 (11) |
| Current housing | Own home | 80 (16) |
| Military Characteristics | |  |
| Rank | Enlisted | 15 (3) |
|  | Non-Commissioned Officer^†^ | 70 (14) |
|  | Commissioned Officer^‡^ | 15 (3) |
| Prior active component or regular service | Yes | 90 (18) |
| # of Deployments to Iraq or Afghanistan (n=16)^§^ | 0 | 15 (3) |
|  | 1 | 45 (9) |
|  | 2 or more | 20 (4) |
| Firearm Characteristics | |  |
| Gun Type Currently in Household |  |  |
| Rifle | Yes | 45 (9) |
|  | No | 35 (7) |
|  | N/A | 20 (4) |
| Handgun | Yes | 75 (15) |
|  | No | 5 (1) |
|  | N/A | 20 (4) |
| Shotgun | Yes | 40 (8) |
|  | No | 40 (8) |
|  | N/A | 20 (4) |
| Number of Guns currently in household | < =2 | 30 (6) |
|  | 3-5 | 25 (5) |
|  | 6+ | 25 (5) |
|  | N/A | 20 (4) |
| Gun locked | Some of time or never | 60 (12) |
| Guns and ammo stored separately | All of the time | 50 (10) |
| Own gun locks | Yes | 40 (8) |
| Use gun locks | Some or none of the time | 25 (5) |
|  | All of the time | 15 (3) |
|  | N/A | 60 (12) |

*includes legally separated

^†^ not mutually exclusive

^‡^ includes Warrant Officers (n=3)

^§^Operation Enduring Freedom/Operation Iraqi Freedom/Operation New Dawn: 1/7/01 to date of interview

N/A: References questions not asked to women who indicated gun ownership but did not have currently in household.
